# Synthesis of nanobelt-like 1-dimensional silver/nanocarbon hybrid materials for flexible and wearable electronics

**DOI:** 10.1038/s41598-017-05347-4

**Published:** 2017-07-10

**Authors:** Joong Tark Han, Jeong In Jang, Joon Young Cho, Jun Yeon Hwang, Jong Seok Woo, Hee Jin Jeong, Seung Yol Jeong, Seon Hee Seo, Geon-Woong Lee

**Affiliations:** 1 Nano Hybrid Technology Research Center, Creative and Fundamental Research Division, Korea Electrotechnology Research Institute (KERI), Changwon, 51543 South Korea; 2 Department of Electro-Functionality Material Engineering, University of Science and Technology (UST), Changwon, 51543 South Korea; 3 Institute of Advanced Composite Materials, Korea Institute of Science and Technology (KIST), Eunha-ri san 101, Bondong-eup, Wanju-gun, Jeolabuk-do 55324 Republic of Korea

## Abstract

Most synthetic processes of metallic nanostructures were assisted by organic/inorganic or polymeric materials to control their shapes to one-dimension or two-dimension. However, these additives have to be removed after synthesis of metal nanostructures for applications. Here we report a straightforward method for the low-temperature and additive-free synthesis of nanobelt-like silver nanostructures templated by nanocarbon (NC) materials via bio-inspired shape control by introducing supramolecular 2-ureido-4[1H]pyrimidinone (UPy) groups into the NC surface. The growth of the Ag nanobelt structure was found to be induced by these UPy groups through observation of the selective formation of Ag nanobelts on UPy-modified carbon nanotubes and graphene surfaces. The synthesized NC/Ag nanobelt hybrid materials were subsequently used to fabricate the highly conductive fibres (>1000S/cm) that can function as a conformable electrode and highly tolerant strain sensor, as well as a highly conductive and robust paper (>10000S/cm after thermal treatment).

## Introduction

Low dimensional metallic nanostructures have received considerable attention due to their potential applications in printed electronics, wearable electronics, catalysis and sensors^1–13^. Flexible and stretchable electrodes can be produced via direct coating or fibre spinning with flexible nanocarbon (NC) materials such as carbon nanotubes (CNTs) and graphene nanosheets for soft electronics applications. Their real-world application, however, has so far been limited by the low electrical conductivity of NC materials after solution processing. This problem can potentially be overcome by using metal nanostructures, with silver (Ag) nanocubes^14–16^, nanoplates^17–20^, nanorods^21^, nanowires^22, 23^, nanobelts^24–27^, nanofilaments^28^, and branched nanocrystals^29, 30^ having already been achieved using stabilizers and ionic species in solution to help control the shape and size. Reduction agents also play an important role in shaping these silver nanostructures by altering the reduction kinetics.

Hybrid materials produced from NC and metals have also been investigated in relation to their catalytic properties and electrical conductivity^31–40^, but most studies have focused on decorating NC surfaces with metal nanoparticles for use in catalysis, sensors, and anti-bacterial applications. Oxidized CNTs and graphene oxide (GO) nanosheets have been used to induce the heterogeneous nucleation of metal nanoparticles, but this approach provides little opportunity to create anisotropic structures because of the fast rate of nucleation and growth. This means that additives must be used to control the final nanostructure, which increases the cost of commercial production, and so there is a need to develop a way of controlling crystal growth by modifying NC materials with special functional groups to eliminate the need for other additives.

In biologically controlled mineralisation, protein or peptide molecules can control the structure of an inorganic material by triggering specific crystal growth. In a mollusc shell, for example, chitin plays a major role in the mineralisation of CaCO_3_ by forming a framework for other macromolecules and, in some cases, can even contribute to controlling polymorphism^41^. Microorganisms and viruses are also capable of functioning as binding sites for metal ions and nucleation sites for metal nanomaterials^42, 43^. The use of biomineralisation can therefore provide a greener and cheaper approach to synthesizing inorganic or metallic materials at ambient conditions. Inspired by this, we herein report that synthesis of one-dimensional (1D) nanobelt-like Ag nanostructures hybridized with NC materials can be achieved at room temperature (RT) by NC-mediated nucleation in a media containing a reducing solvent and reducing agent (hydrazine hydrate). This was achieved by functionalizing the NC surfaces with 2-ureido-4[1H]pyrimidinone (UPy) groups to provide multiple points of interaction with silver ions, and by controlling the reduction kinetics. The UPy-mediated growth of Ag nanobelt structures was demonstrated by observing the selective growth of Ag nanostructures on UPy-modified NC surfaces. The synthesized Ag nanobelt/NC hybrid materials were subsequently used to fabricate stretchable conductive fibres and conductive paper-like sheets for use in flexible and wearable electronics. This bio-inspired synthesis process not only allows for room-temperature synthesis of hierarchical NC/metal materials that can be utilized in flexible electronics but can also minimize the use of expensive metal nanomaterials.

## Results

### Synthesis and characterisation of NC/Ag nanobelt hybrid materials

**Figure 1:**
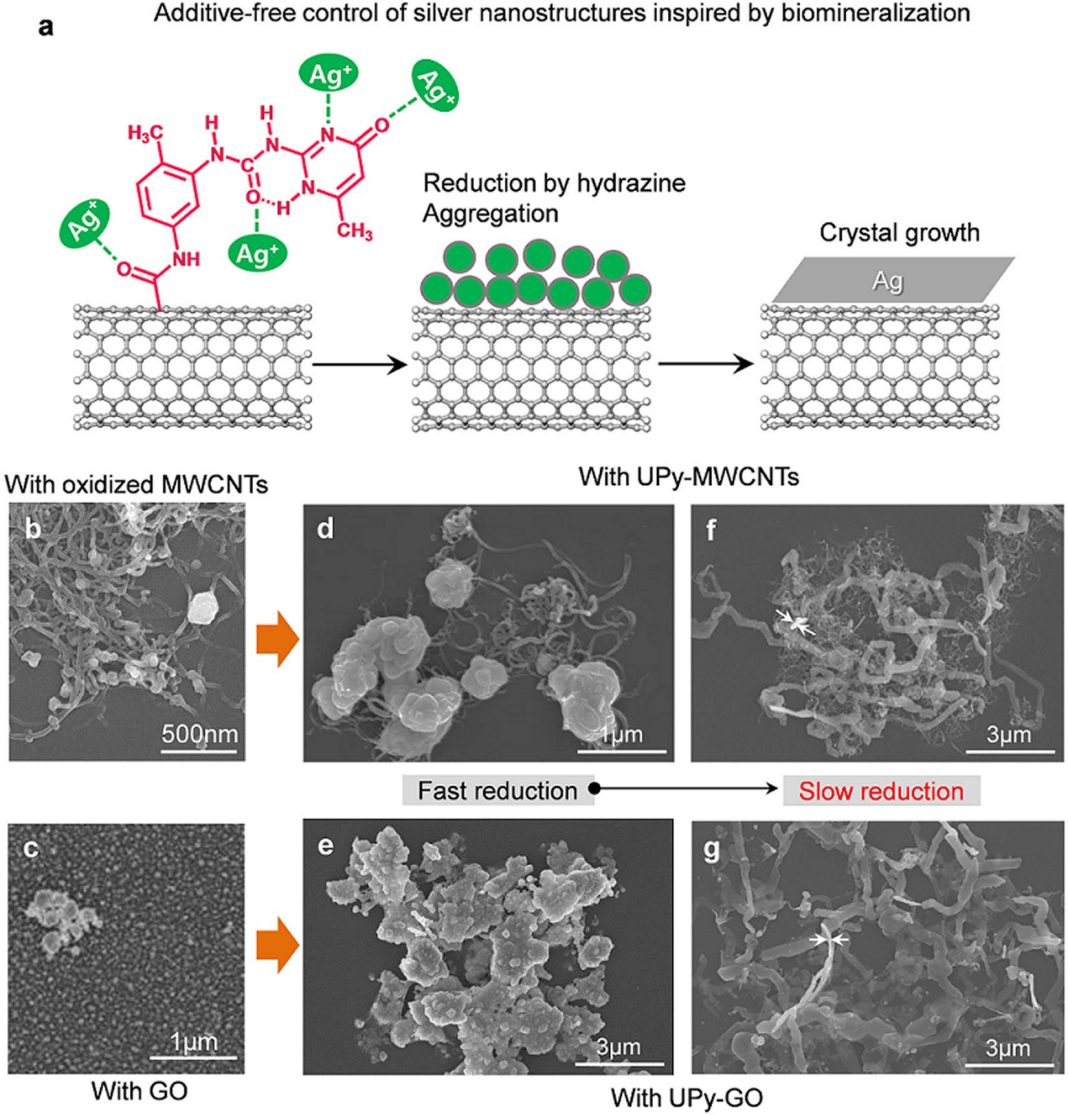
Biomineralisation-inspired synthesis of 1D nanobelt-like Ag structures. (**a**) Schematic for additive-free control of silver nanostructure synthesis templated by NC materials functionalized with 2-ureido-4[*1H*]pyrimidinone (UPy) groups. (**b–g**) FESEM images of silver particles with oxidized MWCNTs (**b**) and GO nanosheets (**c**), and (**d**–**g**) in the presence of (**d**,**f**) UPy-MWCNTs and (**e**,**g**) UPy-GO nanosheets by varying the reduction rate. Arrows in f and g indicate vertically aligned nanobelts showing their thickness.

In microorganisms, metal ions are bound by microbial cells that provide preferential nucleation sites for metal nanomaterial growth. As various thiol, hydroxyl, carboxyl, imidazole, amino, guanidine and imino functional groups have been demonstrated to have a high affinity for binding metal ions^44^. NC materials were functionalized with supramolecular UPy groups that can interact with multiple silver ions (Ag^+^). For this, multi-walled CNTs (MWCNTs) and GO nanosheets functionalized with carboxylic acid or hydroxyl groups were interacted with 2(2-methyl-5-isocyanatobenzylaminocarbonylamino)-6-methyl-4[1H]-pyrimidinone (UPy synthon) to introduce UPy groups on their surface and edges^45–47^. The UPy synthon was synthesized from toluene diisocyanate (TDI) and 2-amino-4-hydroxy-6-methyl-pyrimidine (AHMP) (Figure S1), and the UPy groups introduced onto the NC surface are hypothesized to induce the assembly of Ag nanoparticles and growth of anisotropic Ag nanostructures (Fig. 1a). Moreover, these UPy moieties help the NC materials to disperse in polar solvents, as they provide a large number of hydrogen bonding moieties, even in the presence of ionic species^48^.

Parameters that can affect the morphology of Ag nanoparticles include the choice of reduction agent and concentration, the feeding rate, temperature, and additives such as stabilizers or complexing agents. In this study, hydrazine hydrate and dimethylformamide (DMF) were used as a chemical reduction agent and liquid media for Ag nanostructure formation, respectively^49, 50^. As a control experiment, Ag particles were also synthesized in the presence of silver nitrate (AgNO_3_) and hydrazine in DMF without any other additives at 25 °C. As expected, this produced aggregated Ag microparticles (Figure S2), because the nucleation and growth of the Ag particles were not controlled by any complexing agents. Moreover, in the presence of oxidized NC materials, Ag nanoparticles were decorated on their surfaces as shown in Fig. 1b and c. This demonstrates the need for additives to control the growth of a specific Ag nanostructure, as has been previously reported^10–29^, and so the focus here was on controlling the metal nanostructure without additional stabilizers by using NC materials functionalized with UPy groups (UPy-NC materials).

As the nucleation and growth of crystals is an important part of controlling their structure, the crystallisation of Ag in the presence of UPy-NC was investigated by varying the feeding rate of the hydrazine hydrate reducing agent at 25 °C. Figure 1d and e present representative morphologies of the Ag nanostructures obtained from AgNO_3_ solutions at UPy-MWCNT and UPy-GO concentrations of 0.5 and 0.1 g/L, respectively. Note that the fast reduction of Ag^+^ triggered the formation of micron-sized Ag particles in the presence of UPy-MWCNT and a two-dimensional Ag plate in the presence of UPy-GO, as there was insufficient time for the Ag nanoparticles to assemble during nucleation and growth. However, a notable transition in shape to a 1D-like Ag nanobelt structure was observed when the feeding rate of the hydrazine was slowed to 0.42 ml/min in a 25 mL reactor for 60 min. Thus, at a suitable concentration of 0.2 M AgNO_3_ and 0.01 M hydrazine, Ag nanobelts can be grown in the presence of UPy-NCs, as shown in Fig. 1f and g. Furthermore, when a reducing agent is added, the nucleation of Ag nanostructures occurs at NC surfaces with UPy moieties. It was observed that Ag nanostructures are formed on NC surfaces during the early stages of hydrazine addition, which may be attributed to the large amount of Ag ions interacting with UPy groups (Figure S3). Synthesized Ag nanobelts were less than 50 nm in thickness (Fig. 1f,g and Figure S4).

**Figure 2:**
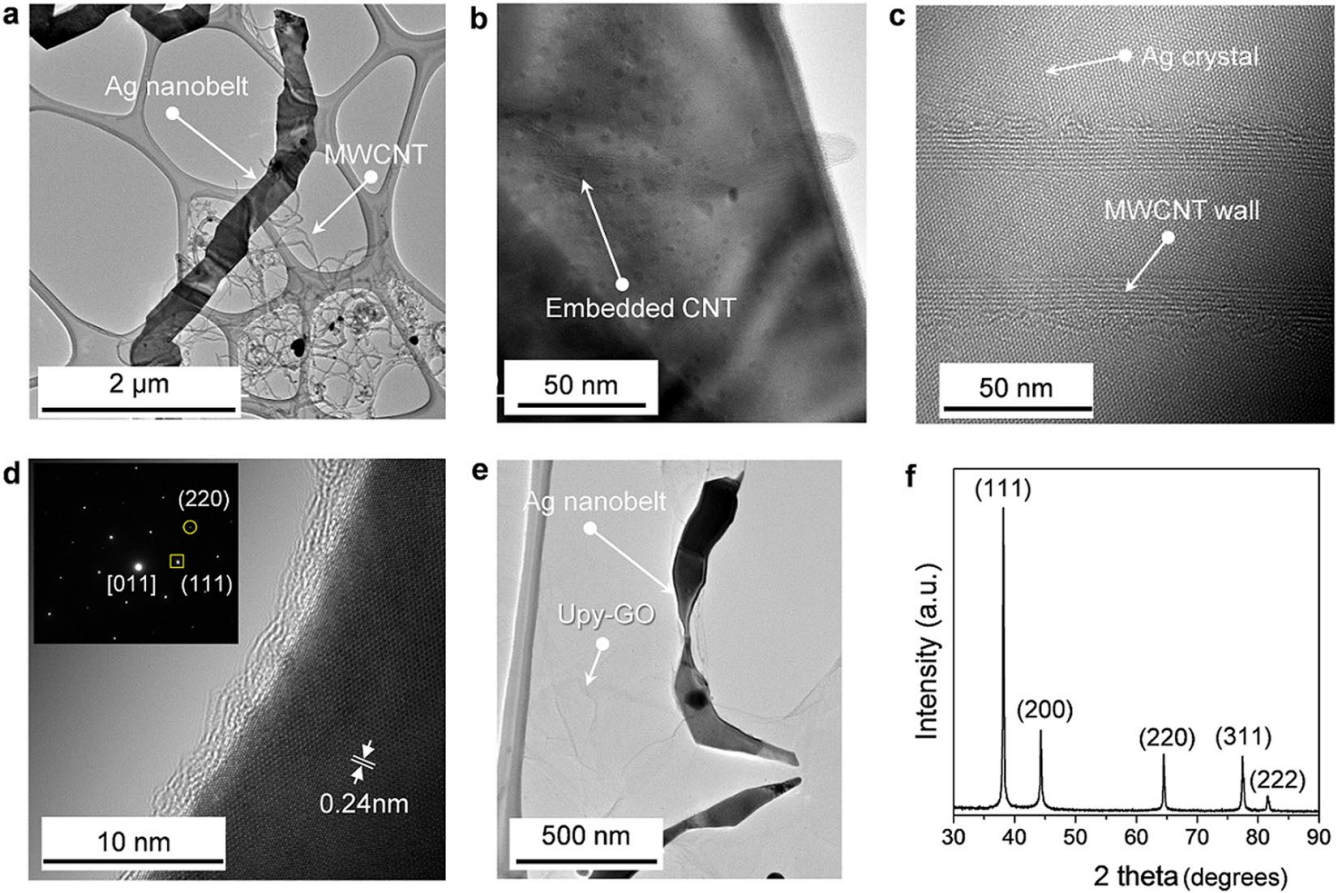
Structural characterisation of synthesized Ag nanobelt/NC hybrid materials. (**a–e**) TEM image of (**a**–**d**) UPy-MWCNT/Ag nanobelt and (**e**) UPy-GO/Ag nanobelt hybrid materials. (**f**) XRD pattern of Ag nanobelt structures.

Figure 2a shows a typical transmission electron microscope (TEM) image of Ag nanobelts grown with the assistance of UPy-MWCNTs. The high magnification TEM images in Figs 2b,c, S5 and S6 show that MWCNTs are embedded in this Ag nanobelt structure, which means that Ag nucleation was initiated on the surface of the UPy-MWCNTs. The crystallinity of these structures was further investigated using selected area electron diffraction (SAED) (see the inset of Fig. 2d for a typical pattern) and HRTEM (Fig. 2d), through which two sets of spots were identified based on their *d*-spacing. The circled spot corresponds to a *d*-spacing of 0.144 nm and can be attributed to {220} reflections, while the inner spots (squared) have a *d*-spacing of 0.24 nm and represent formally {111} reflections of face-centered cubic (FCC) Ag nanobelts^24, 25^. A spacing of 0.24 nm for the planes perpendicular to the edge is likely associated with the {111} reflection, indicating the diffraction pattern of Ag nanobelt has [011] zone axis. The Ag nanobelts on UPy-GO produce similar TEM images, as shown in Fig. 2e. The X-ray diffraction (XRD) pattern of these Ag nanobelts also display similar reflection characteristics to cubic Ag, indicating that they are composed of pure silver. The existence of NC in these hybrid materials was confirmed by Raman spectroscopy (Figure S7).

**Figure 3:**
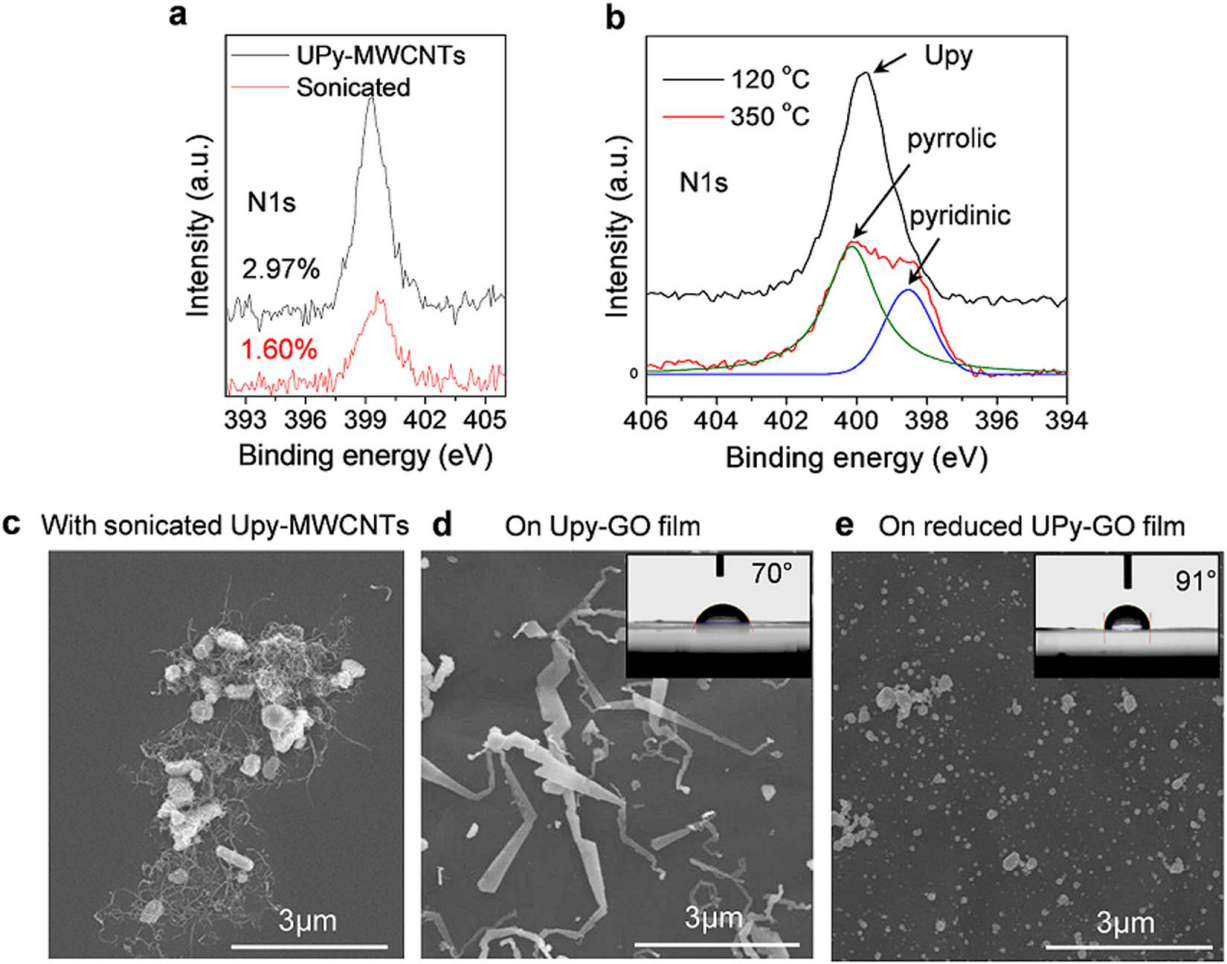
Evidence of NC-templated crystallisation of Ag nanobelt structures. **(a**,**b**) N1s XPS plots of UPy-MWCNTs (before and after sonication) and UPy-GO films after thermal treatment at low (120 °C) and high (350 °C) temperatures. (**c**) Typical FESEM image of Ag nanoparticles with sonicated UPy-MWCNTs synthesized by solution reduction of Ag^+^ ions. (**d**) FESEM image of Ag nanobelt structures on UPy-GO sheet. (**e**) FESEM images of Ag nanostructures on thermally-treated UPy-GO sheet. Inset images in (**d**) and (**e**) show water droplet images and water contact angles on corresponding GO surfaces.

To further demonstrate the UPy-induced nucleation of Ag nanostructures, an attempt was made to intentionally detach the UPy groups from the NC surfaces, as shown schematically in Fig. 3a. To this end, the UPy-MWCNT/DMF solution was ultrasonically treated at high power (500 W) and centrifugally washed prior to Ag synthesis, whereas the UPy-GO paper was thermally treated to detach UPy groups. This detachment of UPy groups was confirmed by X-ray photoelectron spectroscopy (XPS) analysis, with the nitrogen concentration of the MWCNTs being reduced from 2.97 to 1.6 at.% (Fig. 3b and Figure S8). The presence of pyridinic and pyrrolic nitrogen peaks in the XPS spectra of GO following thermal treatment (Fig. 3c) which indicates that this process causes oxygen atoms to be replaced by nitrogen, and so could be used to fabricate N-doped graphene nanosheets. The subsequent synthesis of Ag particles was the same regardless of whether sonicated UPy-MWCNTs or thermally-treated UPy-GO paper was used. Figure 3d–g clearly shows the distinct change in the morphology of the synthesized Ag/NC structures after detachment of UPy groups from the UPy-NC materials. This is similar to the case without any additive, in that Ag micro particles were synthesized in the presence of sonicated UPy-MWCNTs (Fig. 3d). Moreover, nanobelt-like Ag nanostructures were developed on UPy-GO paper (Fig. 3e), but not on the thermally-treated UPy-GO surface (Fig. 3f). This is in stark contrast to the synthesis of Ag nanoparticles on GO (Fig. 3g), as reported in the literatures^37, 40^. Furthermore, the presence of UPy molecules without NC templating did not trigger the formation of anisotropic silver structures (Figure S9). It is therefore believed that supramolecular UPy groups immobilized on NC surfaces is responsible for triggering the nucleation and growth of nanobelt-like 1D Ag structures in the absence of any other additives.

### Flexible electrode application

**Figure 4:**
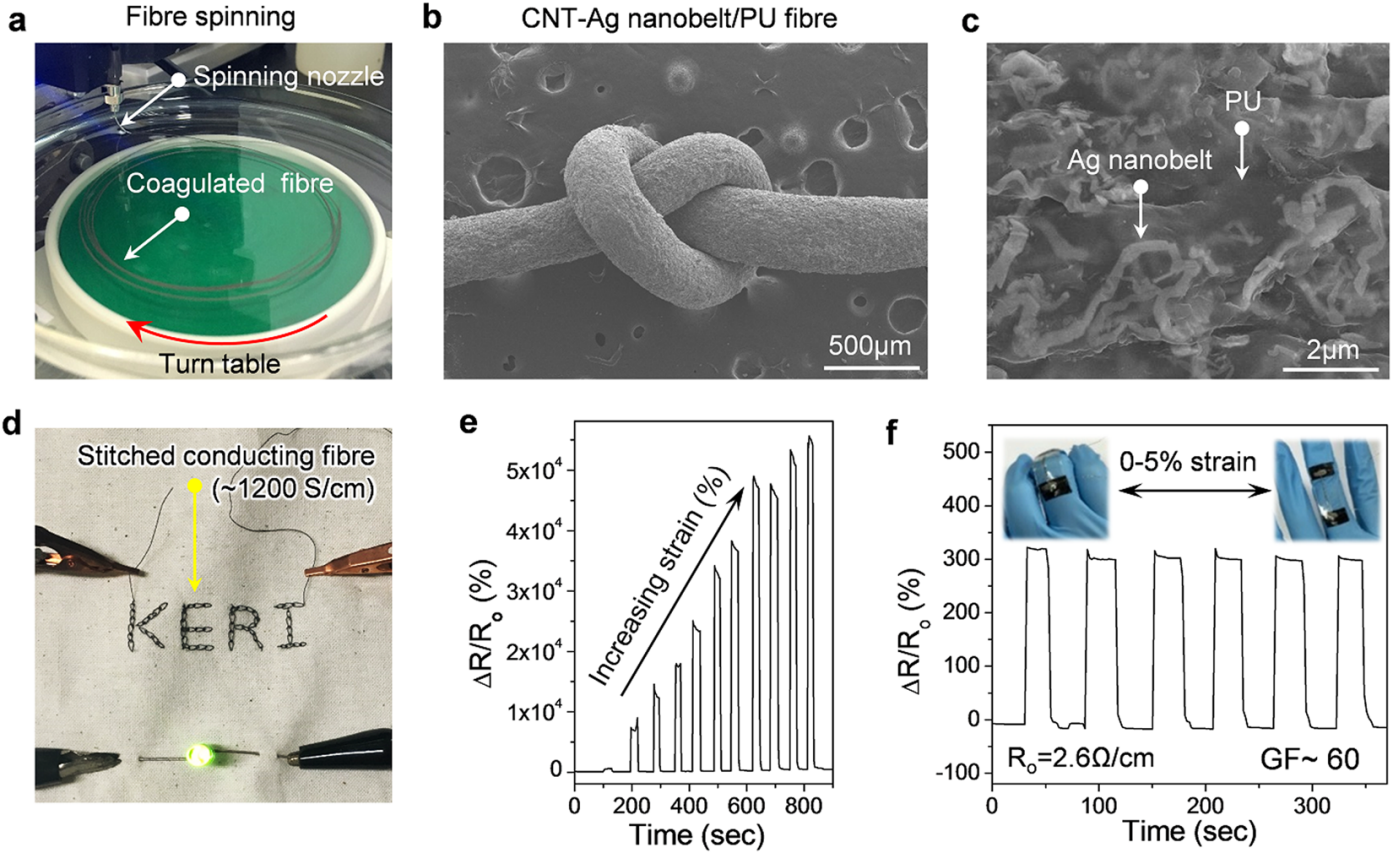
Fabrication of conducting fibres with UPy-MWCNT/Ag nanobelt hybrid materials. (**a**) Photograph of fibre spinning using spinning nozzle and turn table. (**b**,**c**) FESEM image of (**b**) CNT-Ag nanobelt/PU composite fibre and (**c**) its surface. (**d**) Lighting of LED lamp through connection with a stitched conducting fibre. (**e**) Change in resistance of conducting fibre containing 15 wt.% UPy-MWCNT/Ag nanobelt hybrid materials with increasing strain. (**f**) Strain sensor behaviour of conducting fibre on finger (gauge factor ~60).

After that, we tried to demonstrate the potential application of the NC/Ag nanobelts as flexible or stretchable electrodes, UPy-MWCNT/Ag nanobelts were first used as a conducting filler to produce a conductive fibre via solution spinning. To achieve this, a polyurethane (PU) elastomer binder material was dissolved in DMF containing UPy-MWCNT/Ag nanobelts to produce a spinning dope, which was then spun in methanol as a coagulation solvent (Fig. 4a). The fact that this spun fibre could be tied into a knot (Fig. 4b) indicates which means that it could be used in fabric applications. Figure 4c shows a FESEM image of the fibre surface, in which Ag nanobelts can be clearly observed on the surface due to the discharging effect of the metal by electron beam. This fibre exhibited a high electrical conductivity of ~1000S/cm, even when a volume fraction of UPy-MWCNT/Ag nanobelts (about 15 vol.%) was used (Figure S10) due to the fact that the 1D structure of the MWCNTs and Ag nanobelts assists percolation. The use of PU elastomer as a matrix material also means that these conductive fibres are stretchable, and as shown in Fig. 4d, could be easily stitched onto textile to provide stable power to a LED lamp at low voltage (3 V). More importantly, the change in the electrical properties of the composite fibres with strain was found to be dependent on the amount of filler used. That is, at low filler contents of less than 25 vol.%, the resistance of the fibre was sensitive to strain, whereas a highly loaded fibre was electrically stable when strain was applied (Figure S11). Figure 4e shows a typical plot of the relative change in the resistance of a fibre containing 15 vol.% of UPy-MWCNT/Ag nanobelts when the applied strain was increased from 5 to 15%. This UPy-MWCNT/Ag nanobelts/PU strain sensor exhibited a highly tolerant sensitivity with a single monofilament, as indicated by the gauge factor (GF), even at high strain. Furthermore, the resistance increased by over 30000 times at 10% strain and recovered at zero strain, which means that these conducting fibres are very sensitive to physical strain as shown in Fig. 4e. This sensitivity may be due to the belt-like structure of the MWCNT/Ag hybrid material, as well as the high chemical affinity between the hybrid materials and PU matrix. We therefore believe that conductive fibres fabricated with MWCNT/Ag nanobelt hybrid materials could be used in a textile strain sensor. To support this, Fig. 4f shows the reversible strain sensing of a fibre attached onto a finger, in which we see that it produces a high reliability (i.e., a high GF of about 60).

**Figure 5:**
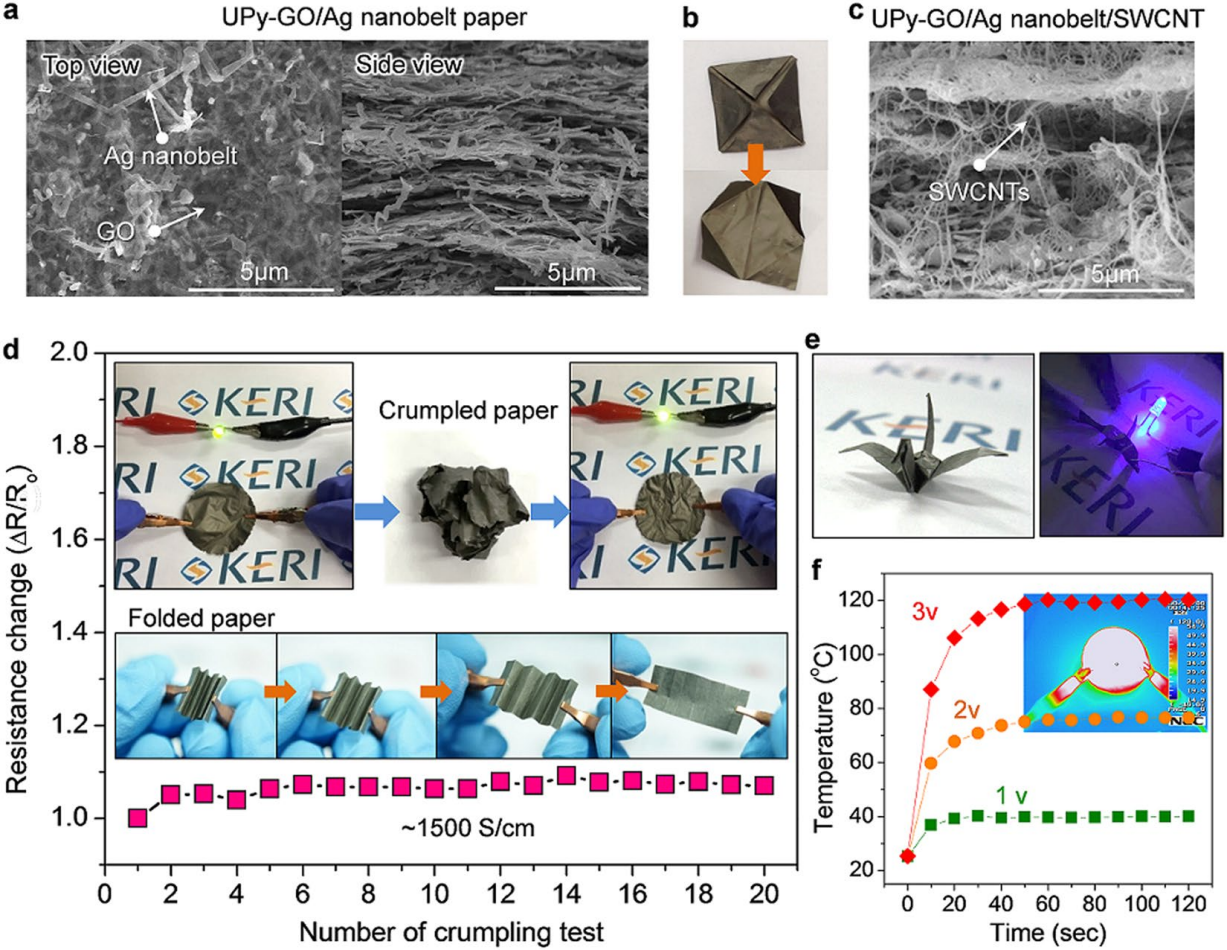
Fabrication of paper-like conducting sheets with UPy-GO/Ag nanobelt hybrid materials. (**a**) Top and side view FESEM images of filtrated conducting paper. (**b**) Photograph of folded UPy-GO/Ag nanobelt paper. (**c**) Side view FESEM image of GO/Ag nanobelt/SWCNT paper. (**d**) Change in resistance of GO/Ag nanobelt/SWCNT paper during crumpling test. Inset images show the stability of the conductive paper after crumpling and folding. (**e**) Photographs of a folded-paper crane and LED lighting with one. (**f**) Thin film heating behaviour of conductive paper with varying supplied DC voltage.

Inspired by the rigid CaCO_3_ layer and organic protein layers of abalone shells, which provide excellent mechanical performance, a highly conductive paper with UPy-GO/Ag nanobelts was produced via filtration. Figure 5a shows a typical FESEM image of the prepared film, in which Ag nanobelts and graphene nanosheets can be observed. Stacked Ag nanobelts inserted into GO nanosheets are also clearly evident in the side-view FESEM image of the UPy-GO/Ag nanobelt (35/65 v/v) paper. Notably, this paper was not only flexible, but also produced a high electrical conductivity of up to 6 × 10^4^ S/cm (Figure S12). However, as this paper was still unstable when highly folded or crumpled, SWCNTs functionalized with UPy groups were added to the pre-filtered solution, as shown in Fig. 5c. This made it possible to obtain a more robust and highly conductive paper (~1500 S/cm) (Fig. 5d), from which a folded-paper crane (Fig. 5e) was created and used to connect a LED lamp to can be lighten at a very low voltage (3 V) power source. It is worth noting that the electrical resistance of this paper did not change, even after 20 crumpling or folding tests (Fig. 5d). The electrical performance of this conductive paper was further demonstrated by using it to make a high-performance film heater, with could provide heating of over 100 °C at a very low DC voltage of 3 V, even after crumpling. It was also found that the current flow when folded was very similar to that of pristine paper, which clearly shows the high mechanical stability of the UPy-GO/Ag nanobelt/SWCNT hybrid paper. Based on these results, we believe that these newly synthesized Ag nanobelt/NC hybrid materials have great potential for application as a conductive material in flexible or textile electronics.

## Discussion

Bio-inspired synthesis is a promising strategy for achieving high-performance, cost-effective production of nanostructured hybrid materials by allowing precise control over crystal structure using supramolecular molecules at RT. In this study, silver nanostructures were modulated by templates made from conductive NC materials that were functionalized with supramolecular functional groups to provide a high affinity for Ag^+^ ions. It was demonstrated that this approach allows 1D-like Ag nanobelt structures to be synthesized in the presence of UPy-NC materials via slow reduction at RT. The one of advantage of this strategy is that it is easy to control the Ag structure without the need for complexing agents that would have to be removed after synthesis. Evidence for this NC-templated synthesis was provided by detaching the UPy groups from the NC surfaces. The Ag nanobelt/CNT hybrid materials were subsequently used to create highly conductive (>1000S/cm) and stretchable fibres that can be used as an electrode or strain sensor. Highly conductive paper-like sheets were also achieved using Ag nanobelt/graphene hybrid materials, and these displayed high stability when folded or crumpled. It is hoped this investigation will create new opportunities for the development of novel NC/nanometal hybrid materials that can be used as conformable electrodes and sensors in wearable electronics.

## Methods

### Synthesis of NC materials functionalized with UPy groups

Synthesis of 2(2-methyl-5-isocyanatobenzylaminocarbonylamino)-6-methyl-4[1H]-pyrimidinone (UPy synthon) was carried out according to the following procedure. First, 1.4 mol of toluene diisocyanate (purchased from Aldrich) was added to 0.7 mol of 2-amino-4-hydroxy-6-methylpyrimidine (purchased from Aldrich), and then heated to 100 °C for 16 h. Anhydrous hexane (2000 mL) was then added and the resulting precipitate was filtered, thoroughly washed with hexane, and then dried at 50 °C under reduced pressure. The MWCNTs were produced by chemical vapour deposition (Hanwha Nanotech) and functionalized with carboxylic acid using a mixture of sulphuric acid and nitric acid (7:3 v/v) at 50 °C for 24 h. Single-layered graphene oxide (GO) nanosheets were synthesized via the oxidation of graphite using a modified Hummers method, followed by exfoliation in water using bath sonication for 30 min.

The UPy groups were introduced onto the NC materials via a coupling reaction with UPy synthon and NCs functionalized with carboxylic acid groups (NCs-COOH). For this, 2.0 g of NCs-COOH was first dispersed in 800 mL of DMF by bath sonication for 1 h under argon purging. The UPy groups were then attached by adding 6.1 g of UPy synthon to the NC suspension, followed by heating to 50 °C for 24 h. Any unreacted UPy synthon molecules were removed by centrifugal washing and vacuum filtration.

### Synthesis of UPy-NC/Ag nanobelt hybrid materials

In a typical synthesis, a 0.1 wt.% UPy-NCs solution in DMF was prepared by bath sonication for 10 min, after which 2 mL of AgNO_3_ (purity 99.8%) solution (0.1 M) was added. This mixture was stirred for a further 10 min to homogenize the reactant, and then 10 mL of hydrazine hydrate (0.01 M) was slowly added to the reactor by a syringe pump. Following reaction at RT for 10 h, the resulting NC/Ag hybrid materials were recovered by washing and centrifugal sedimentation.

### Application of conducting fibres and paper-like conducting sheets

To fabricate the conducting fibres, spinning dopes were prepared by mixing 1 g of UPy-MWCNT/Ag nanobelt powder with 10 wt.% PU solution in DMF using a rotary mixer. The as-prepared conducting pastes were then injected at 10 mL min^−1^ through 22-guage (nominal inner diameter: 0.4 mm) needles into rotating methanol with varying coagulation times. Paper-like conducting sheets were fabricated by filtering UPy-GO/Ag nanobelt hybrid material solutions with a concentration of 150 mg/L.

### Characterisation

The surface and cross-sectional morphology of each sample were imaged by field-emission scanning electron microscopy (FE-SEM, HITACHI S4800). Their structural characteristics were investigated by Transmission electron microscopy (TEM) and selected-area electron diffraction (SAED) experiments which performed on a Titan G2 60–300 (FEI, USA) at an accelerating voltage of 80 kV. The powder X-ray diffraction (XRD) patterns of NC/Ag nanobelt hybrid materials were obtained by using a Philips PW 3830 X-ray diffractometer with Cu Kα radiation (λ = 1.5418 Å). Confocal Raman spectrometry (NTEGRA SPECTRA, NT-MDT) with an excitation wavelength of 532 nm was used to confirm the co-existence of materials. The chemical composition of the NC materials was assessed by XPS using a Multilab2000 (Thermo VG Scientific Inc.) spectrometer with Al K radiation as the X-Ray excitation source. The quantity of organic moieties in each synthesis step was confirmed by thermogravimetric analysis (TA Instruments, TGA Q500). The RT electrical conductivity of the fibres produced was measured by a two-probe resistivity-measuring instrument with uniform 2 mm spacing. The electrical conductivity of the paper sheet was measured by a four-point probe method (Loresta, MCP-T610).

## Electronic supplementary material

Supplementary Information
