# Publisher Correction: Synthesis of nanobelt-like 1-dimensional silver/nanocarbon hybrid materials for flexible and wearable electronics

**DOI:** 10.1038/s41598-018-36014-x

**Published:** 2018-11-30

**Authors:** Joong Tark Han, Jeong In Jang, Joon Young Cho, Jun Yeon Hwang, Jong Seok Woo, Hee Jin Jeong, Seung Yol Jeong, Seon Hee Seo, Geon-Woong Lee

**Affiliations:** 1 Nano Hybrid Technology Research Center, Creative and Fundamental Research Division, Korea Electrotechnology Research Institute (KERI), Changwon, 51543 South Korea; 2 Department of Electro-Functionality Material Engineering, University of Science and Technology (UST), Changwon, 51543 South Korea; 3 Institute of Advanced Composite Materials, Korea Institute of Science and Technology (KIST), Eunha-ri san 101, Bondong-eup, Wanju-gun, Jeolabuk-do, 55324 Republic of Korea

Correction to: *Scientific Reports* 10.1038/s41598-017-05347-4, published online 10 July 2017

The original version of this Article contained a typographical error in the title, where the word “electronics” was incorrectly given as “electroncs”’. This has now been corrected in the PDF and HTML versions of the Article.

