# Supplementary material for: Synthesis of nanobelt-like 1-dimensional silver/nanocarbon hybrid materials for flexible and wearable electronics
Source: Sci Rep. 2017 Jul 10;7:4931. doi: 10.1038/s41598-017-05347-4 (PMC5504065; doi:10.1038/s41598-017-05347-4)
Supplement: Supplementary file 1 — Supplementary Information [file 41598_2017_5347_MOESM1_ESM.doc]

**Supplementary Information**

**Synthesis of nanobelt-like 1-dimensional silver/nanocarbon hybrid materials for flexible and wearable electronics**

Joong Tark Han, ^1,2^ Jeong In Jang,^1^ Jun Young Cho,^2^ Jun Yeon Hwang,^3^ Jong Seok Woo,^1^ Hee Jin Jeong,^1^ Seung Yol Jeong,^1^ Seon Hee Seo,^1^ Geon-Woong Lee^1^

^1^Nano Hybrid Technology Research Center, Creative and Fundamental Research Division, Korea Electrotechnology Research Institute, Changwon 51543, South Korea

^2^Department of Electro-Functionality Material Engineering, University of Science and Technology, Changwon 51543, South Korea

^3^Institute of Advanced Composite Materials, Korea Institute of Science and Technology (KIST), Eunha-ri san 101, Bondong-eup, Wanju-gun, Jeolabuk-do 55324, Republic of Korea

*To whom correspondence should be sent. email: jthan@keri.re.kr

This PDF file includes:

Supplementary Figures S1 to S11


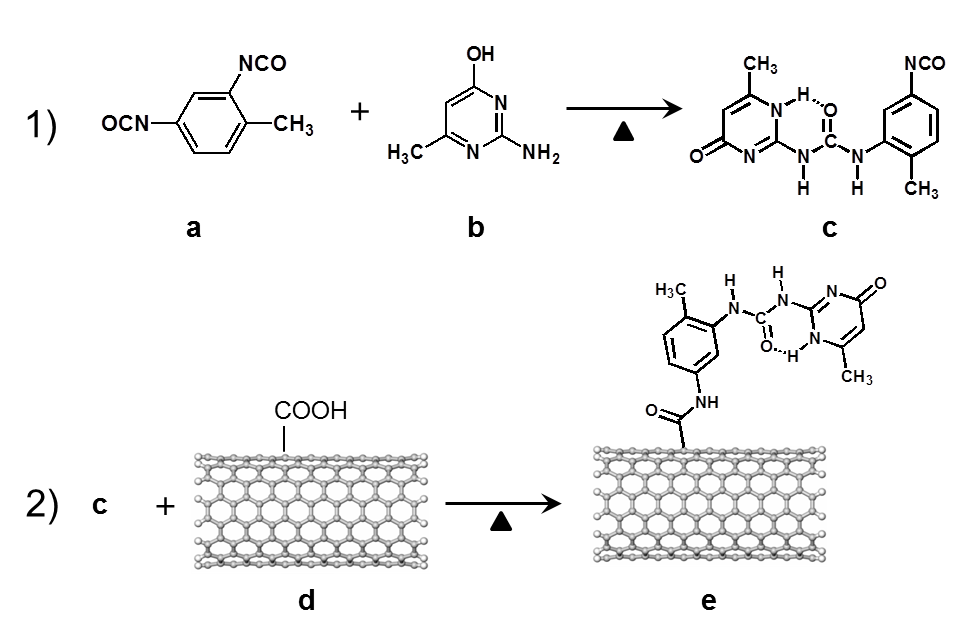


**Supplementary Figure S1 | Schematic of UPy-NC synthesis.** 1. Synthesis of (c) NCO precursor (2(2-methyl-5-isocyanatobenzylaminocarbonylamino)-6-methyl-4[1H]-pyrimidinone) from (a) toluene diisocyanate and (b) 2-amino-4-hydroxy-6-methylpyrimidine at 100 °C for 16 h. 2. Synthesis of (e) UPy-modified NC materials from (c) NCO precursor and (d) NC materials functionalized with carboxylic acid groups at 50 °C for 24 h.


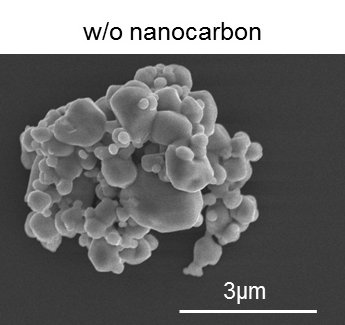


**Supplementary Figure S2 |** FESEM images of silver particles synthesized without NC materials


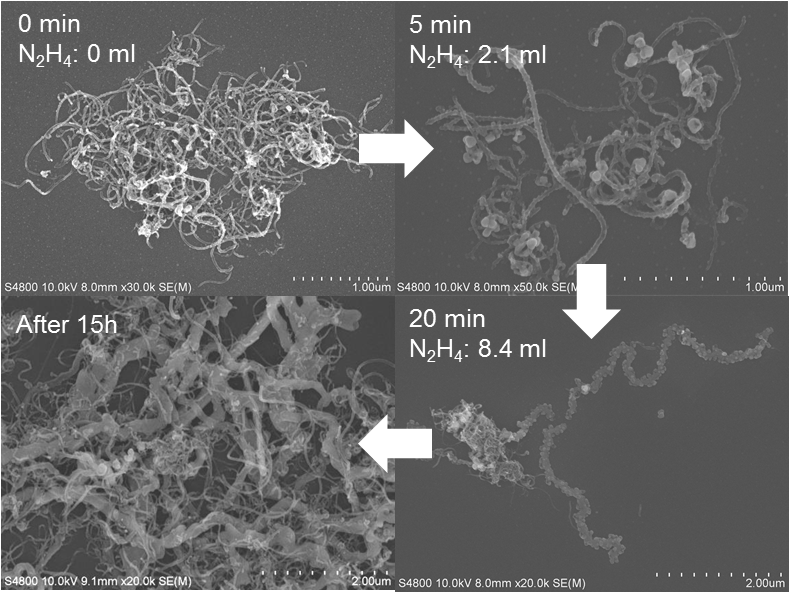


**Supplementary Figure S3 | Observation of Ag crystallisation.** FESEM images of UPy-MWCNT/Ag structures during addition of hydrazine (N_2_H_4_) as a reducing agent at room temperature.

***
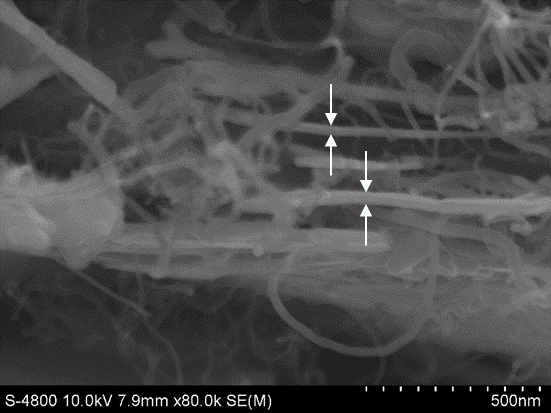
***

**Supplementary Figure S4 |** Side-view FESEM image of the UPy-MWCNT/Ag nanobelt paper showing the thickness of Ag nanobelts indicated by arrows.


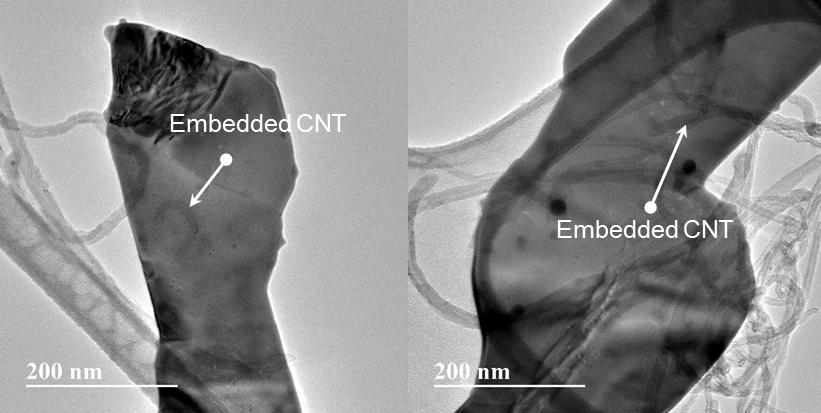


**Supplementary Figure S5 |** TEM images of Ag nanobelts showing embedded CNTs inside Ag structures.


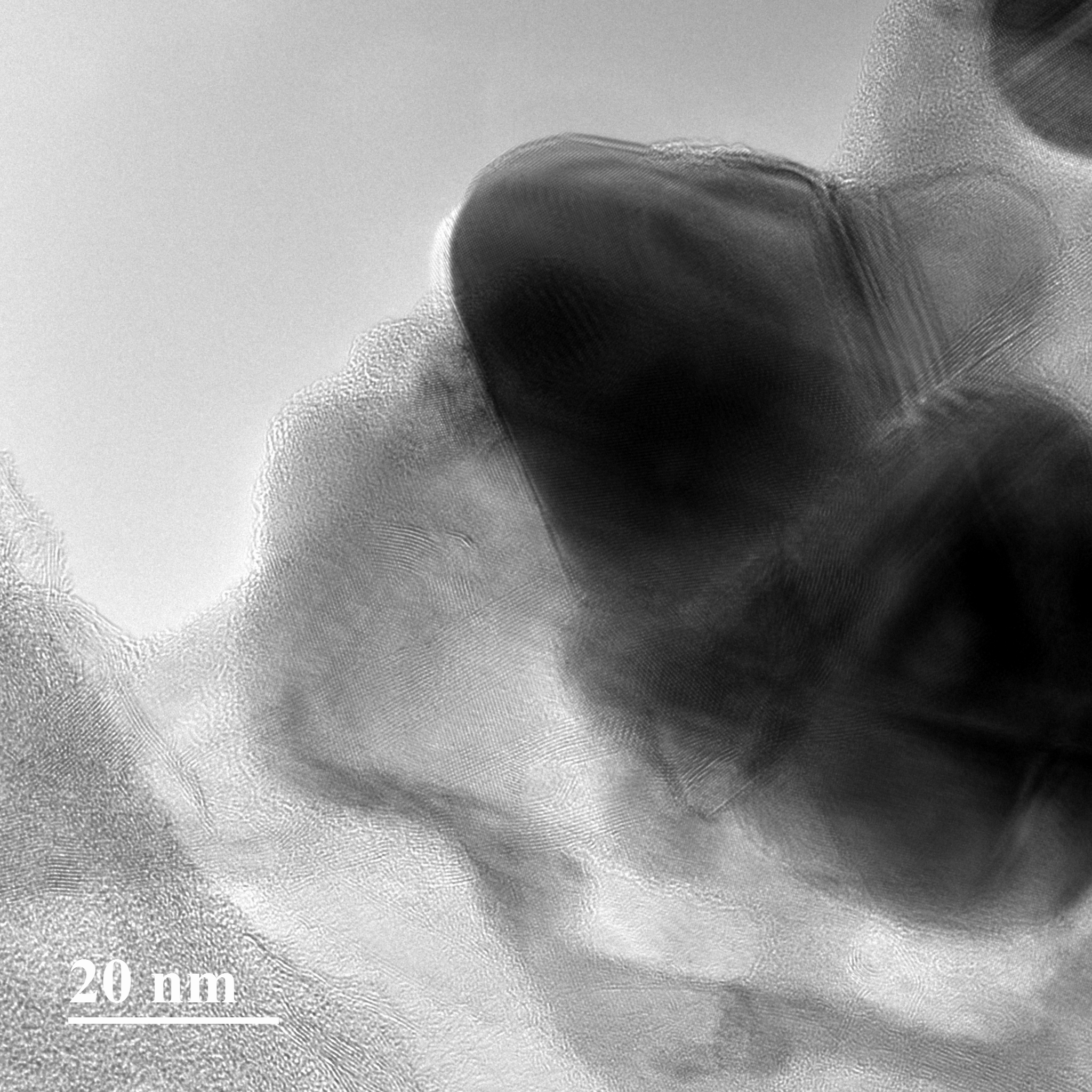

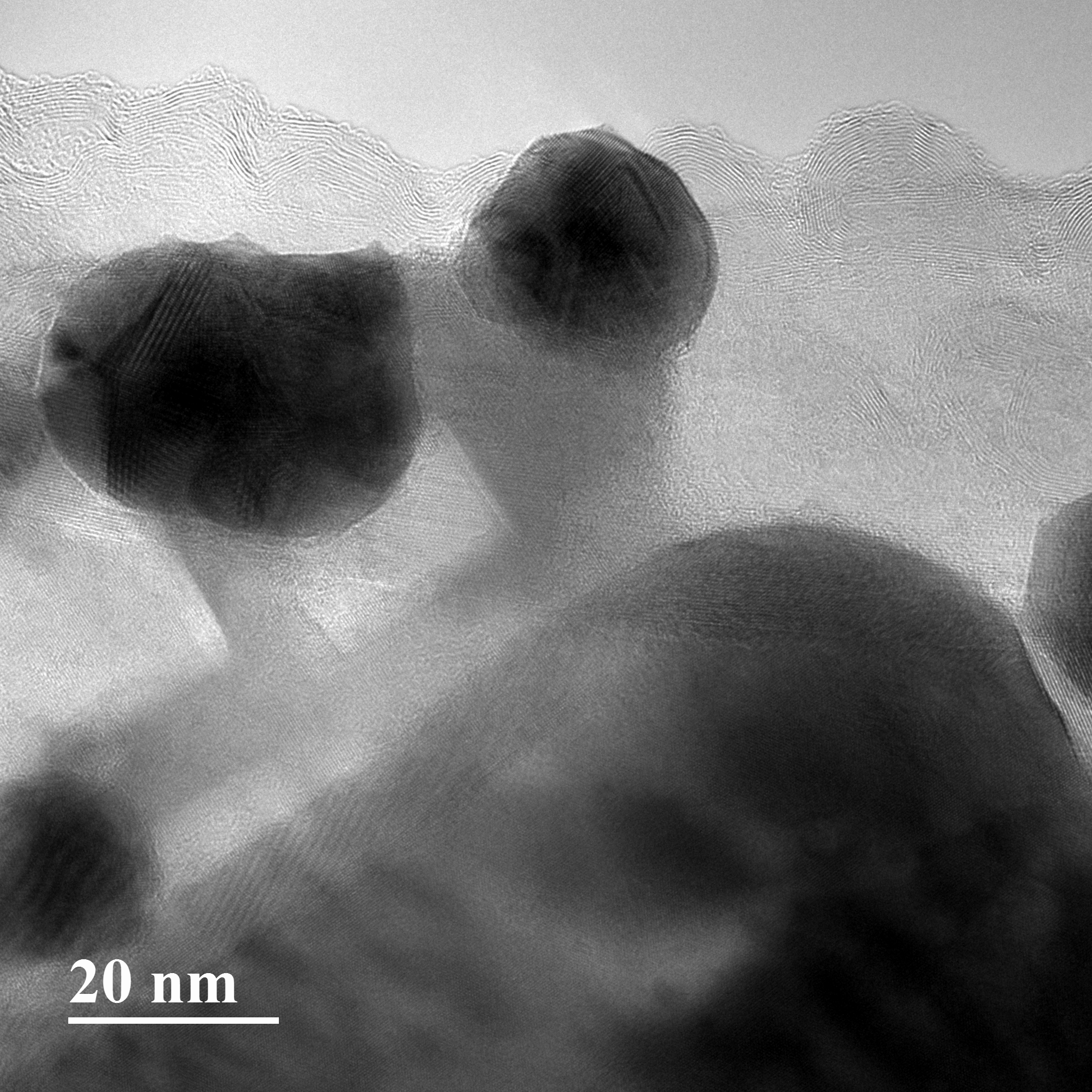


**Supplementary Figure S6 |** TEM images showing the twins in Ag crystals near the embedded CNTs.

**Supplementary Figure S7 | Characterisation of used MWCNTs and synthesized hybrid material.** Raman spectra of pristine MWCNT, MWCNs functionalized with carboxylic acid groups (MWCNT-COOH), MWCNTs functionalized with UPy groups (UPy-MWCNT), and synthesized UPy-MWCNT/Ag nanobelt.


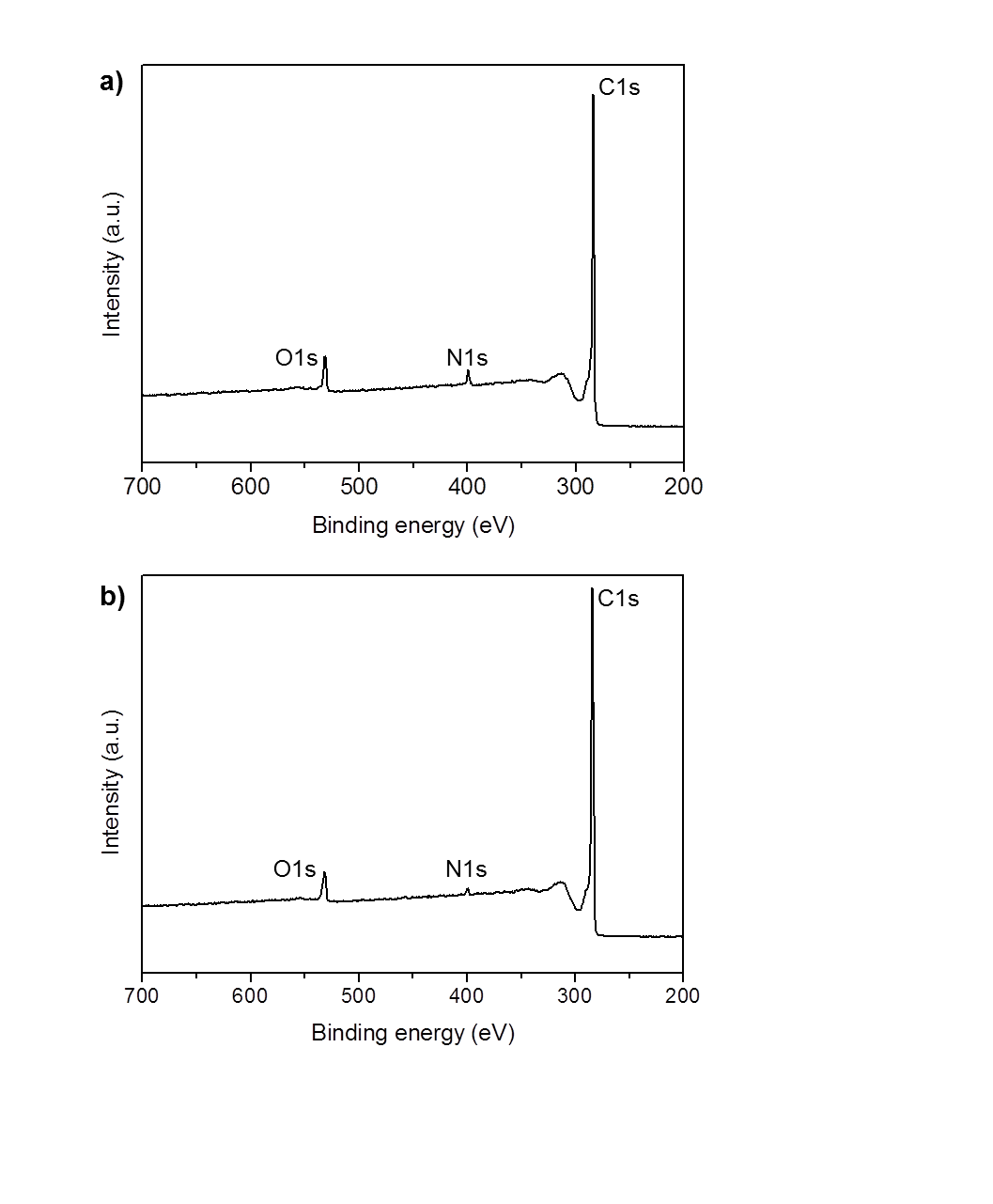


**Supplementary Figure S8 | XPS analysis of UPy-MWCNTs before and after ultrasonic degradation:** **a-b**, Survey scan plots of XPS of UPy-MWCNTs (a) before and (b) after ultrasonication by horn sonicator.


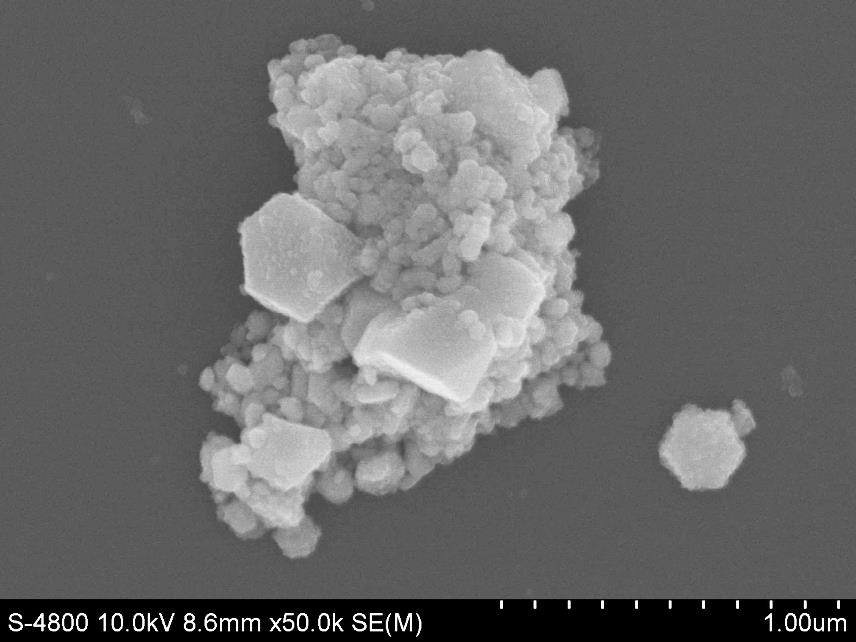


**Supplementary Figure S9 | Characterisation of Ag particles in the presence of UPy synthon.** FESEM image of Ag particles after reduction of AgNO_3_ solution in DMF with UPy molecules not immobilized on NC surfaces.


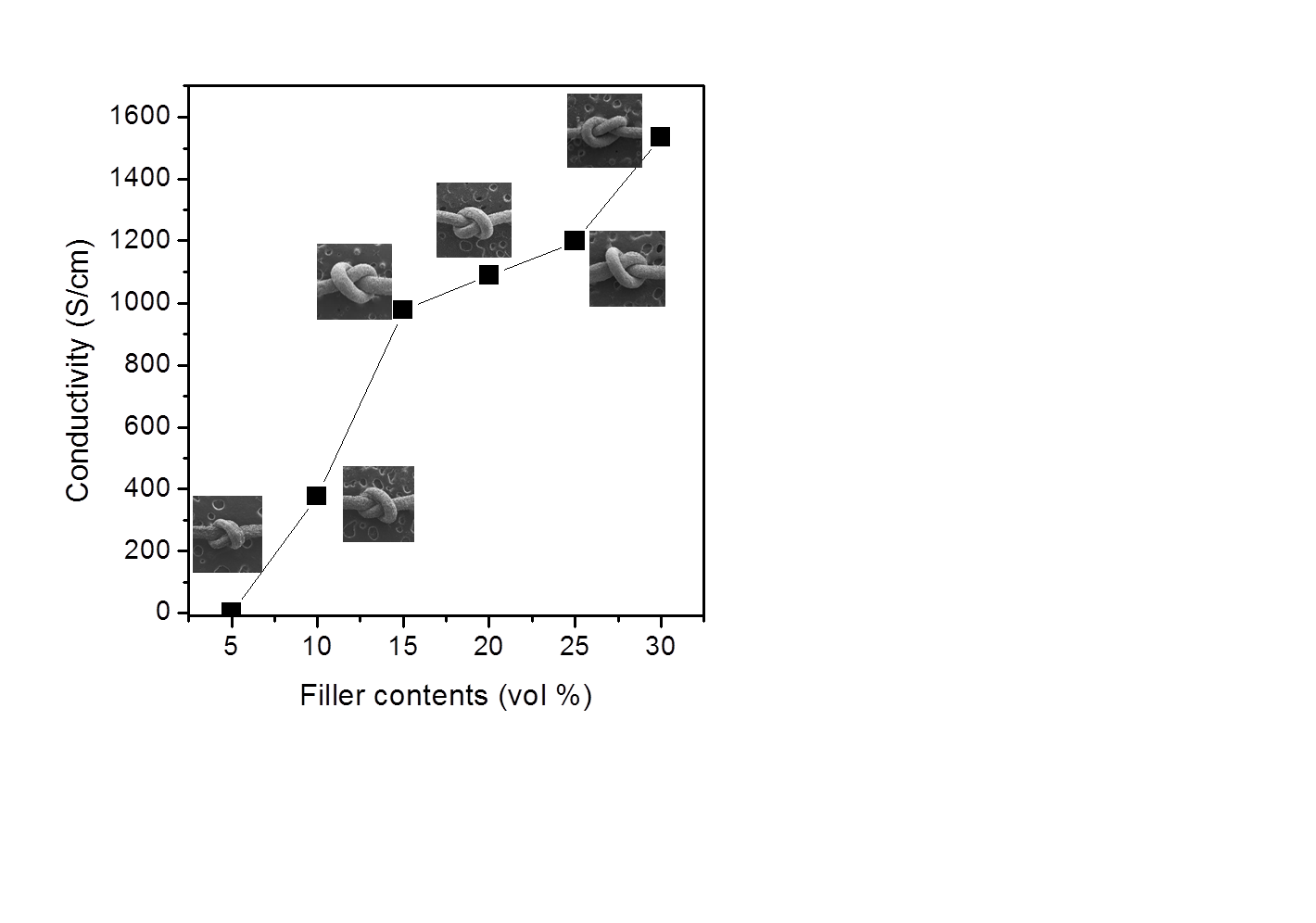


**Supplementary Figure S10 | Electrical properties of spun fibres.** Electrical conductivities of PU/UPy-MWCNT/Ag nanobelt composite fibres. Inset images show FESEM images of knotted conducting fibres.

**Supplementary Figure S11 |** Change in resistance of conducting fibre containing 15 wt.% UPy-MWCNT/Ag nanobelt hybrid materials.


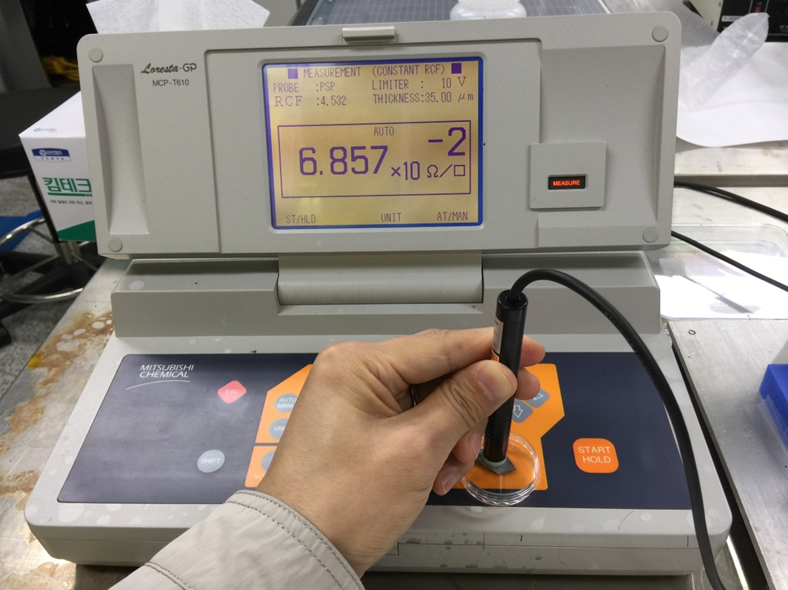


**Supplementary Figure S12 |** Photo image showing the sheet resistance of the UPy-GO/Ag nanobelt paper.
